# Magnetic resonance imaging of acute and chronic atrial ablation injury - a histological validation study

**DOI:** 10.1186/1532-429X-15-S1-O18

**Published:** 2013-01-30

**Authors:** James Harrison, Nick Linton, Sarah A Peel, Amedeo Chiribiri, Anne Yoon Krogh Grøndal, Lars Bloch, Rashed Karim, Steven Williams, Kawal Rhode, Steen Fjord, Matthew Wright, Won Yong Kim, Jacob F Bentzon, Henrik Jensen, Tobias Schaeffter, Reza Razavi, Mark O'Neill

**Affiliations:** 1 Division of Imaging Sciences & Biomedical Engineering, King's College London, London, UK; 2 Department of Cardiology, St Thomas' Hospital, London, UK; 3 Department of Cardiology, Aarhus University Hospital Skejby, Aarhus, Denmark

## Background

Radiofrequency ablation causes a combination of irreversible and reversible atrial injury. Previous publications have suggested that this can be visualized with a combination of T2-weighted (T2W) and late gadolinium enhancement (LGE) CMR. However, signal intensity (SI) thresholds have not been validated in the atrium. This study sought to compare T2W and LGE with histological findings in an animal model of atrial ablation.

## Methods

Under general anesthesia and before ablation, 16 pigs underwent CMR - 3D T2W and LGE (20 minutes after the injection of 0.2ml/kg Gadovist). Next, a linear radiofrequency ablation lesion was created from the SVC to the IVC. The animals were then transferred immediately for post-ablation (acute) CMR according to the same protocol. 8 of the animals were euthanized and the hearts were explanted for macroscopic and microscopic examination. The other 8 animals were recovered for two months before undergoing repeat (chronic) T2W and LGE CMR. These animals were then euthanized and the hearts explanted. The right atria were cut axially at 4mm intervals and the lesion volume estimated. Microscopic sections were stained with hematoxylin and eosin (acute) and Masson's Trichrome (chronic).

SI thresholds from 0 to 15 (at 0.1 intervals) standard deviations (SD) above a reference SI were used to segment the right atrial wall of T2W and LGE images for the detection of ablation injury [the reference SI was defined as LV myocardium for T2W and atrial blood pool (ABP) for LGE]. The resulting segmented volumes of injury were compared with the histological findings in order to find the most realistic threshold.

## Results

Microscopic histology of acute ablation injury confirmed transmural injury with coagulative necrosis, hemorrhage and interstitial edema. Chronic histology demonstrated transmural replacement of normal atrial wall with fibrous scar tissue. The mean acute and chronic volumes of injury were 2.75 ± 1.26 cm3 and 1.51 ± 0.53 cm3 respectively. The SI thresholds that best approximated these volumes were: 2.3 SD above the mean ABP SI for LGE acute; 3.3 SD above the mean ABP SI for LGE chronic; and 14.5 SD above the mean LV myocardium SI for T2 acute. T2 chronic always underestimated the lesion volume.

## Conclusions

This is the first study to define LGE and T2W CMR SI thresholds for defining acute and chronic atrial ablation injury, by comparison with microscopic and macroscopic examination. Extrapolation to human atrial CMR may permit non-invasive assessment of scar burden and position prior to repeat catheter ablation for atrial arrhythmias.

## Funding

British Heart Foundation Clinical Research Training Fellowship; EPSRC/TSB.

**Figure 1:**
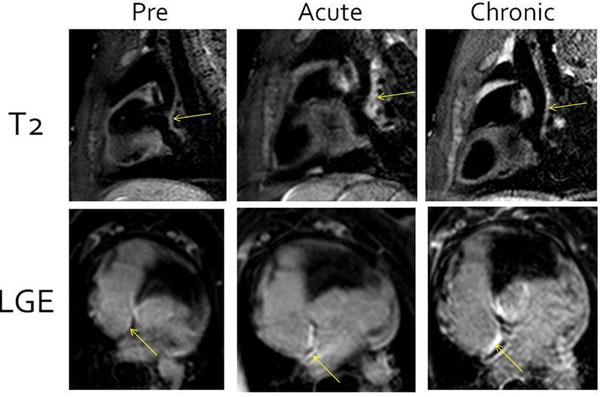
Sagittal T2-weighted images (top row) and axial late gadolinium enhancement images (bottom row) before ablation (pre), immediately after ablation (acute) and after two months (chronic). All images are from the same animal. The posterior right atrial wall (the site of radiofrequency ablation) is indicated with the two arrows.

**Figure 2:**
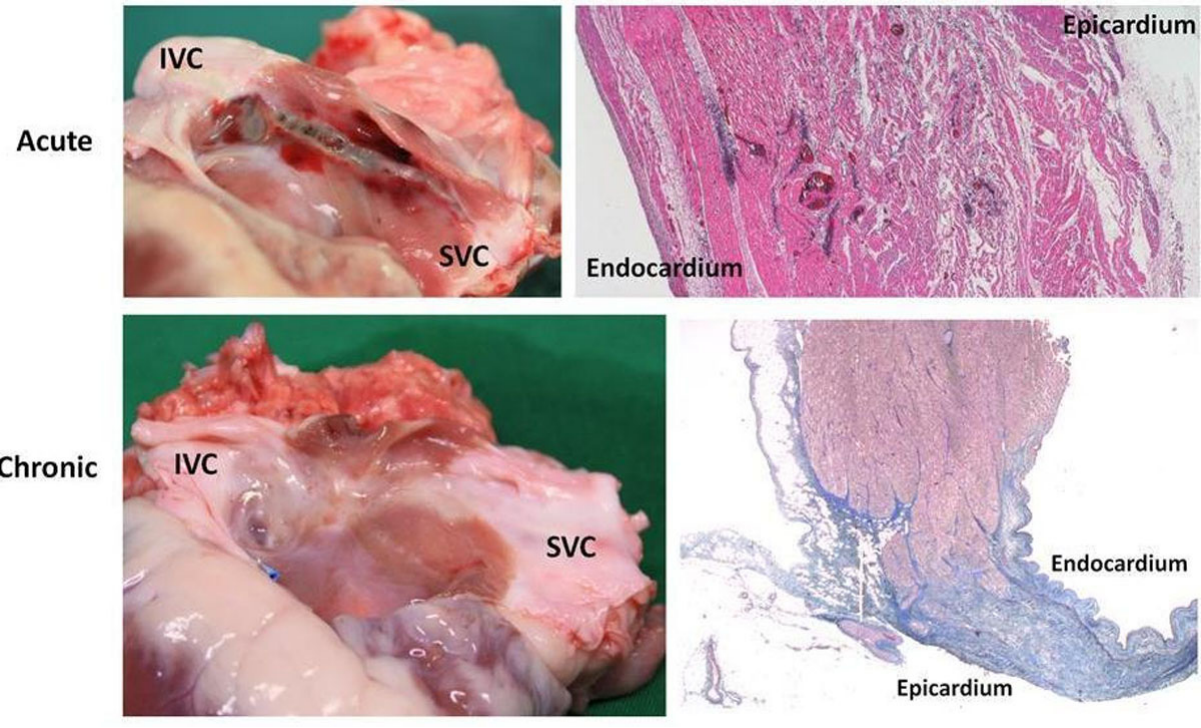
Top row: macroscopic (left) and microscopic (right; stained with hematoxylin and eosin) images of acute atrial ablation injury. Microscopic histology demonstrates transmural injury with coagulative necrosis, hemorrhage and interstitial edema. Bottom row: macroscopic (left) and microscopic (right; stained with Masson’s Trichrome) images of chronic atrial ablation injury. Microscopic histology demonstrates transmural replacement of normal atrial wall with fibrous scar tissue.

